# Deer browsing alters sound propagation in temperate deciduous forests

**DOI:** 10.1371/journal.pone.0211569

**Published:** 2019-02-13

**Authors:** Timothy J. Boycott, Jingyi Gao, Megan D. Gall

**Affiliations:** Biology Department, Vassar College, Poughkeepsie, New York, United States of America; Wildlife Conservation Society Canada, CANADA

## Abstract

The efficacy of animal signals is strongly influenced by the structure of the habitat in which they are propagating. In recent years, the habitat structure of temperate forests has been increasingly subject to modifications from foraging by white-tailed deer (*Odocoileus virginianus*). Increasing deer numbers and the accompanying browsing have been shown to alter vegetation structure and thus the foraging, roosting, and breeding habitats of many species. However, despite a large body of literature on the effects of vegetation structure on sound propagation, we do not yet know what impact deer browsing may have on acoustic communication. Here we used playback experiments to determine whether sound fidelity and amplitude of white noise, pure tones, and trills differed between deer-browsed and deer-excluded plots. We found that sound fidelity, but not amplitude, differed between habitats, with deer-browsed habitats having greater sound fidelity than deer-excluded habitats. Difference in sound propagation characteristics between the two habitats could alter the efficacy of acoustic communication through plasticity, cultural evolution or local adaptation, in turn influencing vocally-mediated behaviors (e.g. agonistic, parent-offspring, mate selection). Reduced signal degradation suggests vocalizations may retain more information, improving the transfer of information to both intended and unintended receivers. Overall, our results suggest that deer browsing impacts sound propagation in temperate deciduous forest, although much work remains to be done on the potential impacts on communication.

## Introduction

Animal communication involves the production of a signal by a sender, the transmission of that signal through the environment, and the detection of that signal by a receiver [1]. The efficacy component of animal signals is determined, in large part, by the transmission properties of the signaling environment [1,2]. The sound propagation characteristics of habitats therefore play a key role in the evolution of vocal signals. Selection pressures often favor acoustic signals that minimize degradation and attenuation; thus enhancing the propagation of signals and optimizing communication in a given environment (Acoustic Adaptation Hypothesis; [3,4]). Changes to the sound propagation characteristics of an environment could, therefore, alter the selection pressures on the efficacy components of signals, driving changes to the structure of signals over time [3–6].

There are two major types of changes that occur as signals propagate through the environment: degradation and attenuation [7]. Degradation is a change in the structure of the signal itself (i.e. a change in fidelity), which may arise in a variety of ways, while signal attenuation is a reduction in signal amplitude that is a product of both spherical spreading and habitat-specific excess attenuation [1,2]. Signal degradation can result from, for example, reverberation and scattering of sound waves by physical impediments in the propagation path and thus sound signals traveling via many different pathways to the receiver. This non-uniformity in propagation path can cause irregular amplitude fluctuations as well as temporal effects such as echoes [5,7]. The extent of degradation and attenuation depends on the interaction between the structural characteristics of the sound signal itself, abiotic factors such as humidity, wind, and temperature, and the structure of the environment (i.e. vegetation structure, ground characteristics) [1,2,8,9].

The effect of vegetation structure and ground characteristics on sound propagation are well known [9–11]. Generally, acoustic signals are subject to more reverberation and scattering in forested habitats than in open habitats [5,11,12]. This is particularly true for higher frequency sounds, which are more likely to be reflected by objects in the environment [13]. This leads to greater fidelity and less attenuation of lower-frequency sounds in forests, due to the long wavelengths of these sounds [11]. In open habitats, however, sound signals are more influenced by air movements which can mask low-frequency sounds and add slow modulations to signals, thus higher frequency and more rapidly modulated signals propagate with greater fidelity and less attenuation than lower frequency signals [8,10,14]. For signals propagating close to the ground, the structure of the ground cover plays an important role in signal propagation. Hard-packed soils can increase the reverberation of signals, while ground cover with irregular porous structure, such as leaf litter, can have a deadening effect leading to very little reverberation [9, 15]. Changes in vegetation structure or ground characteristics are therefore likely to influence the characteristics of sound propagation in forested habitats.

The structure of temperate forests has been changing due to browsing by white-tailed deer (*Odocoileus virginianus*). In the last century, white-tailed deer populations in North America have rapidly increased and have now reached 30 million individuals, largely due to increased foraging, restricted hunting regulations, and the eradication of natural predators [16,17]. The effects that deer have on birds and other taxa are primarily mediated through browsing-induced changes in understory vegetation composition. Deer browsing leads to the gradual eradication of browsing-sensitive plant species from the habitat and a concomitant increase in species that are browse-tolerant [18–20]. Deer browsing can, in some cases, increase species richness by assisting the dispersal of seeds [19,21], selectively browsing on dominant species [22,23] and increasing light availability in the understory [24]. However, deer browsing can also reduce the density and abundance of understory trees and shrubs and increase the coverage of sedges, mosses and bare ground [19,25,26,27]. Reductions in vegetation biomass can also lead to changes in leaf litter composition and soil characteristics [28,29]. The reduction and homogenization of understory vegetation often results in a cascade effect on invertebrate and songbird species, driving population declines [30–35]. Increased deer abundance has been, in particular, associated with a decline in understory-dependent songbirds, by reducing access to food resources and nesting sites and by increasing predation [36–38].

Despite a strong interest in deer browsing and its ecology, its effects on communication remain largely unstudied. Thus, we investigated the effects of deer browsing on sound propagation over short distances using pairs of browsed and unbrowsed plots. Many bird species use short-range contact, courtship, or food-related calls that propagate over these distances [39–42]. As we were interested in first determining whether and how deer browsing would affect sound propagation, we chose to use artificial stimuli that allowed us to tightly control the frequency and temporal modulation of our stimuli, rather than single species exemplars of vocalizations. We used three primary stimuli types, broadband, pure tone, and trilled stimuli, which represent basic components of vocalizations in a variety of taxa. We expected that differences in sound propagation in deer-browsed and deer-excluded habitats would primarily result from differences in vegetation structure between the habitats; however other deer-induced changes (e.g. leaf litter composition, biotic noise spectra) could also play a role. We predicted that sound fidelity would be higher, and sound attenuation would be lower, in deer-browsed habitats compared to deer-excluded habitats, because scattering and reverberation should be reduced with a reduction in vegetation density in deer-browsed habitats. However, these plots were still within forested areas, thus we did not expect an increase in wind-generated masking. More specifically, we predicted that broadband, trilled and high-frequency sounds would propagate with greater fidelity and less attenuation in deer-browsed habitats due to reduced vegetation and thus reduced reverberation. On the other hand, we predicted that low-frequency sounds and tonal stimuli would not differ substantially between deer-browsed and deer-excluded habitats. Finally, we expected that stimuli with a propagation path through understory vegetation would differ more across browsing treatments, than those with a propagation path above the understory due to larger changes in vegetation density due to deer browsing in the understory.

## Methods

### Ethics statement

Our experiments were conducted at the Vassar College Farm and Ecological Preserve (VCFEP), which is owned by Vassar College. No specific permit or authorization was required for this work, as no animals were used in the experiment, nor did the study affect any endangered or protected species. However, all work was coordinated with the manager of the VCFEP to ensure that our work would not interfere with other experiments.

### Study area

All experiments were conducted between June 10^th^ and June 16^th^ 2015 at the Vassar College Farm and Ecological Preserve, a 527-acre preserve surrounded by a suburban residential matrix in Poughkeepsie, New York. The habitat is primarily mixed deciduous forest, interspersed with coniferous tree species. Dominant deciduous species include red maple (*Acer rubrum*), white oak (*Quercus alba*), and white ash (*Fraxinus americana*). At the time of our study the deer population density at the Vassar Farm and Ecological Preserve was estimated to be between 19 and 21 animals per square mile using aerial infrared flyover photography and on-the-ground deer fecal pellet counts methods [43]. Previous estimates showed densities reaching between 40 and 50 individuals per square mile as recently as 2014 [43]. Deer density of this magnitude has previously been shown to have a significant impact on both vegetation structure and avian communities, such as reduced understory vegetation diversity and abundance and decline in forest-songbird species dependent on understory vegetation [31,32,38]. In 2008 three sets of 10 x 10m paired plots were established in temperate deciduous forest to investigate the effects of deer browsing. At each site, one of the two plots was enclosed by a 3m tall fence to exclude deer (hereafter: deer-excluded), while the other had its corner boundaries denoted by stakes, but was otherwise open to deer browsing (hereafter: deer-browsed). Two additional 10 x 10m paired plots were established in 2012. All five sites were between 200 and 1800m apart from one-another, in temperate deciduous forest habitats of the 527-acre preserve. Preliminary analyses suggest that exclusion of deer affected vegetation structure (2015 data: fenced (mean ± S.D.) = 80 ± 26 saplings plot^-1^; unfenced = 11 ± 7 samplings plot^-1^; comparison photos in S1 Fig) [43].

### Stimuli

We used three types of stimuli that are representative of sounds found in acoustic animal signals: pure tones, trills, and white noise [7,44]. All stimuli were generated in PRAAT (ver. 5.3.55; Boersma and Weenik, 2013) at a sampling rate of 44.1 kHz. The 16 pure tones were 0.5s in duration with 0.1ms gating (rate at which stimulus goes from zero to full amplitude) to facilitate the identification of signals on the subsequent recordings and ranged from 0.5 kHz to 8 kHz in 0.5 kHz steps. The tones were separated on the recording by 0.5s silent intervals. The nine trill exemplars consisted of eight 10ms tonebursts with 0.1ms gating of frequencies of 1, 3, or 5 kHz. We created “fast”, “medium” and “slow” trills by adjusting the inter-element interval to 10, 40, or 90 ms respectively. Finally, the white noise was 5s in duration with 0.1ms gating and was bandpass filtered from 0.1 to 9 kHz, to match the frequency spectrum of our playback speaker. The playback speaker did not have a flat frequency response. Therefore, prior to our field experiment, we measured the stimulus voltage to projected amplitude functions at each frequency. We then determined the voltage level to which each stimulus would need to be set such that the stimuli would be projected from the speaker at equivalent amplitudes (as measured with a 1/3 octave band SLM). Thus, the frequency profile of our stimuli in the field was flat.

### General experimental design

We conducted playback experiments in each pair of fenced and unfenced plots at each of the five sampling locations. To maximize the distance over which we could record, the microphone was always placed in one corner of the plot. The corner was randomly selected for each pair (same corner used for the excluded and browsed plot in each pair). The speaker was moved in a straight line across the plot to the opposite corner. Playback experiments were performed twice at each location. One experiment was conducted at 05:00 AM near the dawn chorus and the other was conducted at 10:00 AM. The playback experiment generally took 1.5 hours to complete at each site. The order of which of the two plot types were sampled first was counterbalanced across locations and times of day. At each location we recorded the temperature, average wind speed and humidity with a Kestrel 3000 weather meter at the beginning and at the end of playback experiments. Playbacks were not conducted on days with precipitation or average wind speeds above 2m/s, in efforts to minimize variation in abiotic variables between sampling times.

Playbacks were conducted at three different speaker-microphone height combinations: (1) the microphone and speaker both at a height of 0.75m above the ground (low-low), (2) the microphone at a height of 0.75m and the speaker at a height of 2m above the ground (low-high), or (3) the speaker and microphone both at a height of 2m above the ground (high-high). These were chosen to mimic the propagation path for two birds communicating in the understory, two birds communicating from higher perches, or one bird in the understory receiving a signal from a bird singing from a higher perch [7,45,46]. In all playbacks the microphone was stationary to minimize fluctuations in background noise and only the speaker was moved. We recorded playbacks when the linear distance between the microphone and the speaker was 1, 3, 5, 7, 9, and 11m. In the low-low and high-high height setups, both speaker and microphone were kept horizontal using T-levels and the devices were aligned along the transect using the tape measure. In the low-high height setup, the speaker was kept horizontal using a T-level, but the microphone angle was adjusted to aim directly at the speaker, following a line established with the tape measure. Three replicate trials of the complete playback of the stimuli set were conducted at each distance and height combination. If a loud noise (e.g. airplane flying over, wind gusts, etc.) was observed during a particular distance and height playback trial, that playback trial was terminated and re-recorded once the sound event had passed. We also recorded three minutes of background noise at the beginning and the end of each playback trial.

At the beginning of the experiment we placed a tripod equipped with a T-level (to ensure the tripod was flat) in the corner of the plot (either deer-excluded or deer-browsed). We attached a Pyle PSPL05R digital sound level meter to the tripod. We then placed a Pignose speaker (model 7100) one meter from the sound level meter. The speaker was connected to a Marantz solid state recorder (model #PMD661) that contained the stimuli .wav files. The playback speaker was placed on a custom-built platform equipped with a T-level attached to a tripod. This allowed us to carefully adjust the height and level of the platform. We calibrated the speaker using the sound level meter, adjusting the playback level of the white noise stimulus to 65 dB (dBA weighting and slow integration time). We then replaced the sound level meter with a Sennheiser ME62 microphone (with a windscreen) and K6 powering unit. The microphone was connected to a second Marantz PMD661. The recording level of the Marantz was set such that the voltage produced by the microphone would translate to an amplitude reading of approximately 65 dB in PRAAT and the knob was taped in place to minimize changes in recording level during the experiments. Prior to our field work, we tested this set-up in an IAC audiology booth lined with pyramidal acoustic foam and found that stimuli recorded in this manner were reproduced faithfully (normalized cross-correlation values of > 95%).

### Sound analysis

Playback recordings were transferred from the Marantz to a desktop computer in the lab. The spectrograms of all recordings (dynamic range of 20 dB) were visually inspected to ensure that there were no loud overlapping sounds that could affect our results. We were interested in three main attributes of our sounds: fidelity, amplitude, and attenuation. For stimulus fidelity we used a normalized cross-correlation approach, where a waveform that is identical to a template waveform will have a score of 1 and a waveform that has no relationship to the template waveform will result in a score of 0. This normalized approach removes the effects of amplitude from the cross-correlation, allowing us to focus only on the similarity of waveform shape. For each stimulus we used the recording at one meter (for each height and time of day within a particular enclosure) as the template. We then cross-correlated this template with each recording of that stimulus at each subsequent distance (3 to 11 m). This resulted in cross-correlation at each of five distances for each stimulus within each treatment, height, and time of day combination. We had three replicates of each stimulus at each combination of treatment (deer-excluded vs. deer-browsed), height, time of day, and distance, for which we generated a mean cross-correlation score.

To calculate amplitude and attenuation of our stimuli we first used a custom script in PRAAT to isolate each of the 26 exemplars from the continuous recording. Tones and trills were bandpass filtered at ± 100 Hz from the center frequency of the stimulus. We then used the “Get Intensity” function in PRAAT to determine the overall amplitude of each stimulus. We then calculated a mean amplitude for the three replicates in each condition. We were also interested in the attenuation of the stimuli. Therefore, we calculated the attenuation for each distance by subtracting the amplitude of the stimulus at 1m from the amplitude of the stimulus at each subsequent propagation distance. A mean attenuation was then calculated for the three replicates. We measured the amplitude of the trills including silent periods in between trill elements. Thus, slower trills, with longer silent periods between trill elements of equal amplitude, had lower overall relative amplitudes than faster trills.

Finally, we analyzed our background noise level by bandpass filtering the background noise (a) across the entire frequency range of the stimuli, with a 100Hz buffer on each side (400–8100 Hz) and (b) at each tone center frequency in 200 Hz bands (e.g. 400–600 Hz, 900–1100 Hz, etc.). We then determined the level of each filtered background noise file using the “Get Intensity” function in PRAAT.

### Statistical analysis

All data were analyzed with SAS (version 9.3). We first checked for normality and homogeneity of variances in PROC UNIVARIATE. We analyzed our data with repeated measures mixed model with an autoregressive covariance structure, which resulted in the best model fit according to AIC values. Degrees of freedom were calculated with the Kenward-Rogers algorithm. In each model sampling location was treated as a subject. First, we analyzed the overall background noise level, as well as the spectral profile of background noise in a model that included three independent class variables: frequency, treatment, and time of day. We had three sets of models for the signal propagation data: one set to investigate stimulus fidelity (cross-correlation), one to assess the amplitude of the propagated stimuli, and one set to assess stimulus attenuation. Within each set we ran separate models for each of the three stimulus types: tones, trills and white noise. The between-subject independent variable in each of these models was the treatment (deer-excluded or deer-browsed). The within-subject independent variables were time of day (early or late), speaker-microphone height (low-low, high-high, or low-high), and distance (3, 5, 7, 9, and 11 meters). For the attenuation and fidelity models this resulted in 180 playbacks (60 x 3 replicates) for each of the stimuli. The 1m distance was also included in the amplitude models, for a total of 216 playbacks (72 x 3 replicates) for each of the stimuli. For the tones data set there was an additional independent variable of frequency (0.5–8 kHz) and for the trills data set there were two additional independent variables of frequency (1, 3, or 5 kHz) and trill rate (slow, medium or fast). All interactions between variables were initially included in the models. Non-significant higher-order interactions were removed according to p-value and resulting AIC value. The interaction effects of treatment by distance, treatment by height, treatment by frequency, and treatment by time of day were included in all the models. These interactions were found to be of interest in preliminary analyses, and so were kept constant to allow for comparisons across the models. All subsequent tables and figures present results according to these criteria. To control for environmental variation across trials we included temperature (°C), average wind speed (m/s) and humidity (%) in the models. Significant effects were compared post-hoc with the slice and/or diffs option in the LSMEANS statement. The sound amplitude and sound attenuation models were generally similar in their results; thus, we present only the sound amplitude model here (see S1 Table and S2 and S3 Figs for sound attenuation results). All data can be found in the appendix (S1 Appendix).

## Results

### Background noise

We found that the background noise level did not differ across time of day (F_1,4_ = 0.59, p = 0.49) nor did it differ across treatments (F_1,4_ = 0.03, p = 0.87). Furthermore, we did not find an effect of the time of day by treatment interaction on background noise level (F_1,4_ = 1.08, p = 0.3). The lowest average noise level was found in the early, deer-excluded plots (mean ± S.D. = 36.1 ± 2.85 dB SPL) and the highest average noise level was found in the late deer-excluded plots (mean ± S.D. = 39.1 ± 4.08). The noise levels in the deer-browsed plots were intermediate (early mean ± S.D. = 37.51 ± 3.38; late mean ± S.D. = 37.05 ± 4.56). We found no evidence that the spectral profiles of the noise differed across the day (time of day by frequency: F_15,60_ = 0.18, p = 0.99), across treatments (treatment by frequency: F_15,60_ = 1.34, p = 0.21) or with the time of day by treatment interaction (time of day by treatment by frequency F_15,60_ = 0.3, p = 0.99). However, unsurprisingly, the sound levels did change with the frequency band (frequency: F_15,60_ = 58.82, p < 0.001; S2 Table).

### Sound fidelity

**Fig 1:**
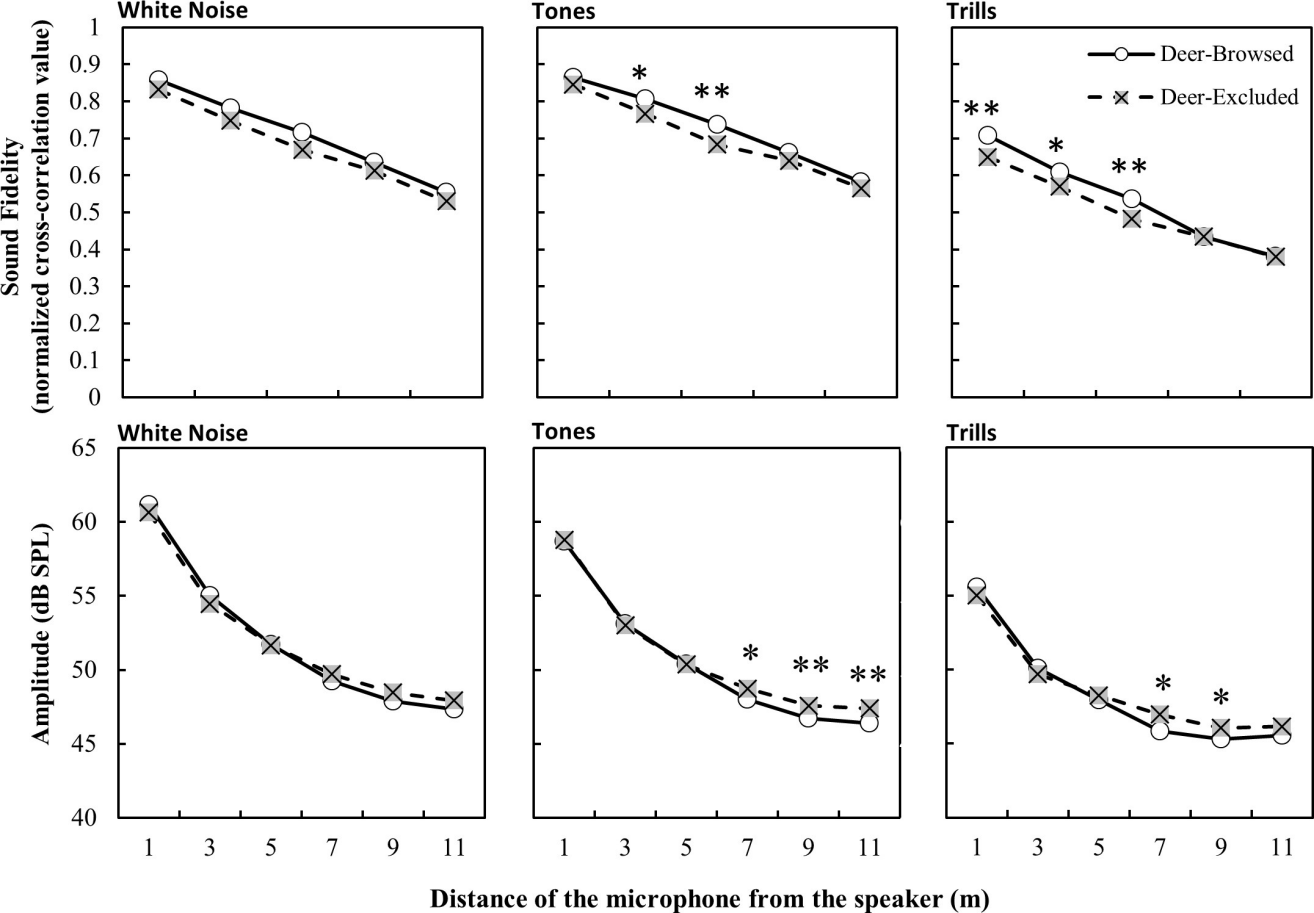
Propagation fidelity and amplitude of stimuli (lsmeans ± S.E.) as a function of distance and browsing treatment. Significant post-hoc differences in least-square means between treatments at each distance are denoted by * p < 0.05, ** p < 0.001.

**Fig 2:**
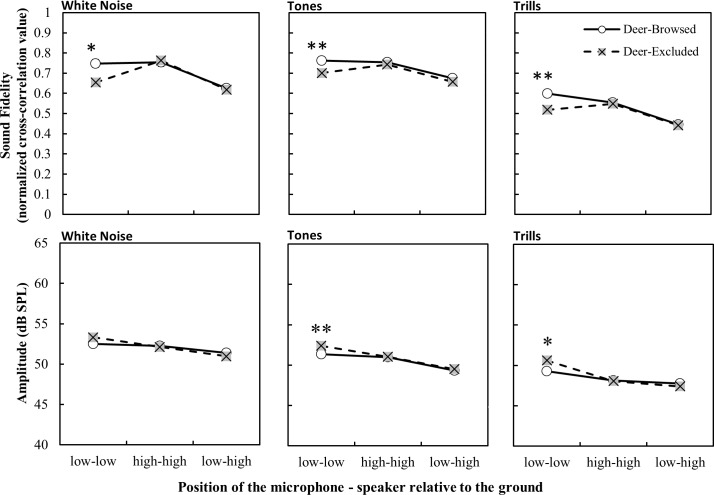
Propagation fidelity and amplitude of stimuli (lsmeans ± S.E.) as a function of playback height and browsing treatment. Low-low indicates both speaker and microphone at 0.75m above the ground; high-high indicates both speaker and microphone at 2m above the ground; low-high indicates the microphone at a height of 0.75m and the speaker at a height of 2m above the ground. Significant post-hoc differences in least-square means between treatments at each height setup are denoted by * p < 0.05, ** p < 0.001.

Average wind speed had a significant effect on the fidelity of all stimuli types, ambient temperature had a significant effect on the fidelity of tone and trill stimuli and relative humidity had no effect (Table 1). We found that sound fidelity was significantly greater in areas with deer browsing than in those without browsing for tones and trills, but we found no difference for the white noise stimuli (Table 1). Fidelity decreased with increasing propagation distance, with stimuli in deer-browsed areas having greater fidelity than stimuli in deer-excluded areas at short propagation distances (Fig 1). Moreover, the height of the propagation path was important for all stimuli, as the difference in fidelity was greatest when both the speaker and the microphone were placed in the understory (Table 1 and Fig 2). However, the propagation fidelity did not differ across treatments when the speaker was placed above the understory.

**Table 1:** Sound fidelity of white noise, tone, and trill stimuli.

Effect:	White Noise			Tones				Trills		
	DF	F-value	p-value	DF	F-value	p-value		DF	F-value	p-value
Temperature	1, 43	0.91	0.3458	1, 654	6.03	0.0143		1, 170	4.84	0.0291
Humidity	1, 35.6	0	0.9503	1, 651	1.27	0.2596		1, 158	0.06	0.8044
Average Wind Speed	1, 83.3	7.01	0.0097	1, 669	52.32	< .0001		1, 219	28.49	< .0001
Treatment	1, 66.3	2.46	0.1215	1, 669	23.94	< .0001		1, 202	6.98	0.0089
Time of day	1, 54.2	3.4	0.0706	1, 660	32.83	< .0001		1, 186	14.04	0.0002
Distance	4, 231	221.41	< .0001	4, 760	293.85	< .0001		4, 445	142.6	< .0001
Height	2, 241	44.82	< .0001	2, 663	72.59	< .0001		2, 178	46.37	< .0001
Frequency	-	-	-	15, 4022	153.55	< .0001		2, 2561	1270.91	< .0001
Trill rate	-	-	-	-	-	-		2, 2118	6998.54	< .0001
Treatment * Time of day	1, 82.8	1.37	0.2457	1, 662	12.94	0.0003		1, 179	1.24	0.2678
Treatment * Distance	4, 246	0.47	0.7596	4, 1169	1.88	0.1116		4, 1540	4.51	0.0013
Treatment * Height	2, 89.4	3.25	0.0433	2, 700	6.99	0.0010		2, 259	6.07	0.0027
Treatment * Frequency	-	-	-	15, 4027	2.36	0.0022		2, 2491	8.36	0.0002
Time of day * Height	+	+	+	2, 716	0.6	0.5500		2, 470	1.3	0.2748
Time of day * Frequency	-	-	-	15, 4023	10.62	< .0001		2, 2395	0.56	0.5695
Distance * Height	+	+	+	8, 753	5.3	< .0001		8, 683	1.72	0.0912
Distance * Frequency	-	-	-	60, 4262	3.36	< .0001		8, 1644	5.74	< .0001
Height * Frequency	-	-	-	30, 4185	16.96	< .0001		4, 2242	37.05	< .0001
Treatment * Trill rate	-	-	-	-	-	-		2, 2192	1.53	0.2171
Time of day * Trill rate	-	-	-	-	-	-		2, 2125	0.46	0.6327
Distance * Trill rate	-	-	-	-	-	-		8, 2465	6.76	< .0001
Height * Trill rate	-	-	-	-	-	-		4, 2396	0.74	0.5647
Frequency * Trill rate	-	-	-	-	-	-		4, 2328	237.14	< .0001
Treatment * Time of day * Height	+	+	+	2, 734	4.48	0.0116		2, 410	3.4	0.0345
Treatment * Time of day * Frequency	-	-	-	15, 4023	3.54	< .0001		2, 2415	2.52	0.0804
Treatment * Height * Frequency	-	-	-	30, 4148	1.84	0.0035		+	+	+
Time of day * Height * Frequency	-	-	-	30, 4146	2.33	< .0001		+	+	+
Distance * Height * Frequency	-	-	-	120, 4279	2.7	< .0001		16, 2495	8.84	< .0001
Treatment * Time of day * Trill rate	-	-	-	-	-	-		2, 2134	0.91	0.4046
Treatment * Height * Trill rate	-	-	-	-	-	-		4, 2130	0.09	0.9860
Time of day * Height * Trill rate	-	-	-	-	-	-		4, 2131	0.05	0.9945
Distance * Frequency * Trill rate	-	-	-	-	-	-		16, 2407	3.61	< .0001
Height * Frequency * Trill rate	-	-	-	-	-	-		8, 2355	3.25	0.0011
Treatment * Time of Day * Height * Trill rate	-	-	-	-	-	-		4, 2130	3.54	0.0069

Weather variable effects, main effects, two-way interactions, three-way interactions and four-way interactions are separated by lines. Variables that are not applicable to a model (e.g. those with frequency for white noise) are indicated by (-). Non-significant higher order interactions removed prior to the final model are indicated by (+).

**Fig 3:**
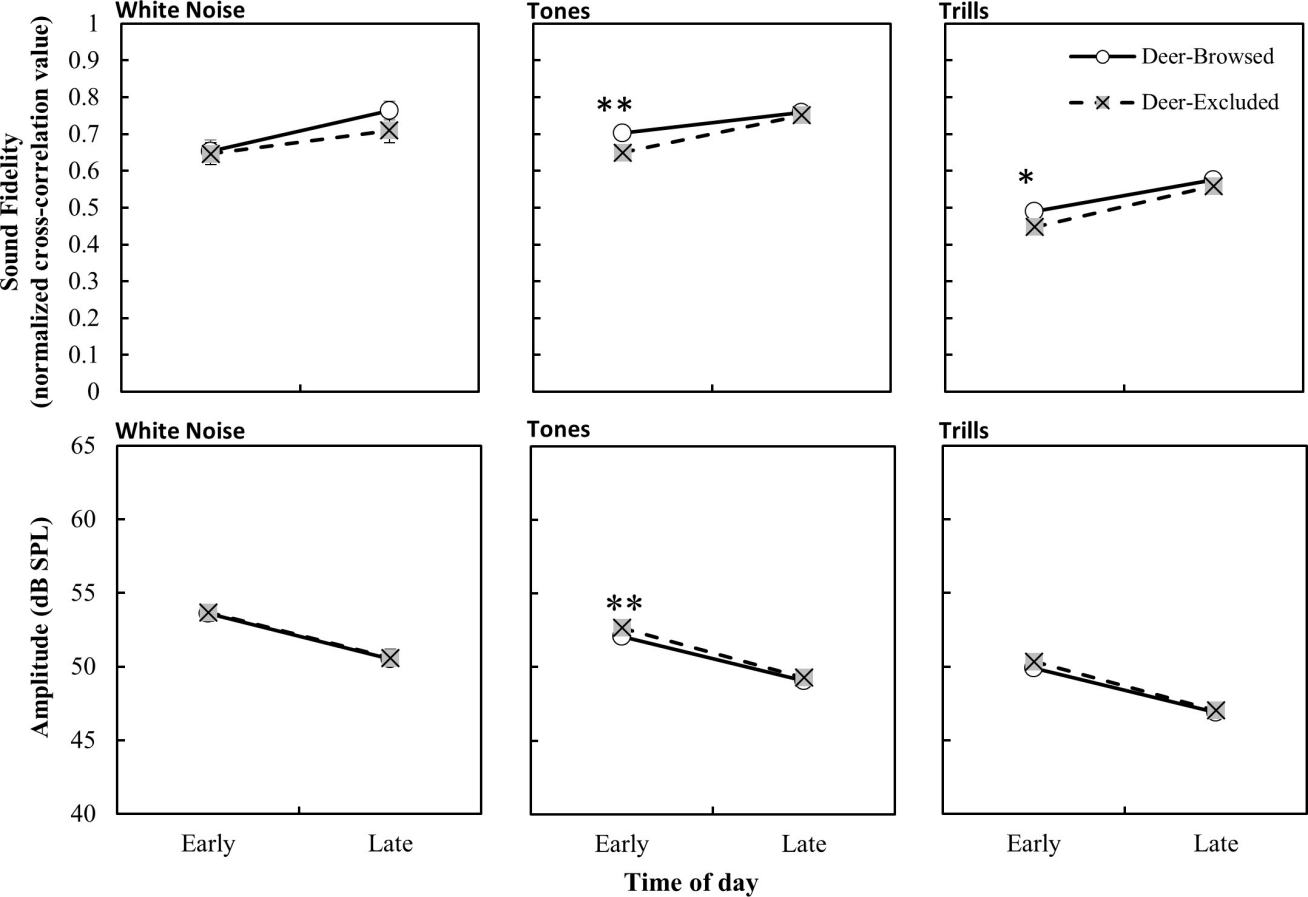
Propagation fidelity and amplitude of stimuli (lsmeans ± S.E.) as a function of time of day and browsing treatment. Early measurements were taken between 0500 and 0700. Late measurements were taken between 1000 and 1200. Significant post-hoc differences in least-square means between treatments at each time of day are denoted by * p < 0.05, ** p < 0.001.

Sound fidelity of tones and trills was greater later in the day (Table 1 and Fig 3) and the effect of deer browsing was more pronounced during the dawn chorus than later in the day, with deer-browsed areas having higher fidelities. (Fig 3). White noise stimuli were not influenced by the treatment by time of day interaction (Table 1 and Fig 3).

**Fig 4:**
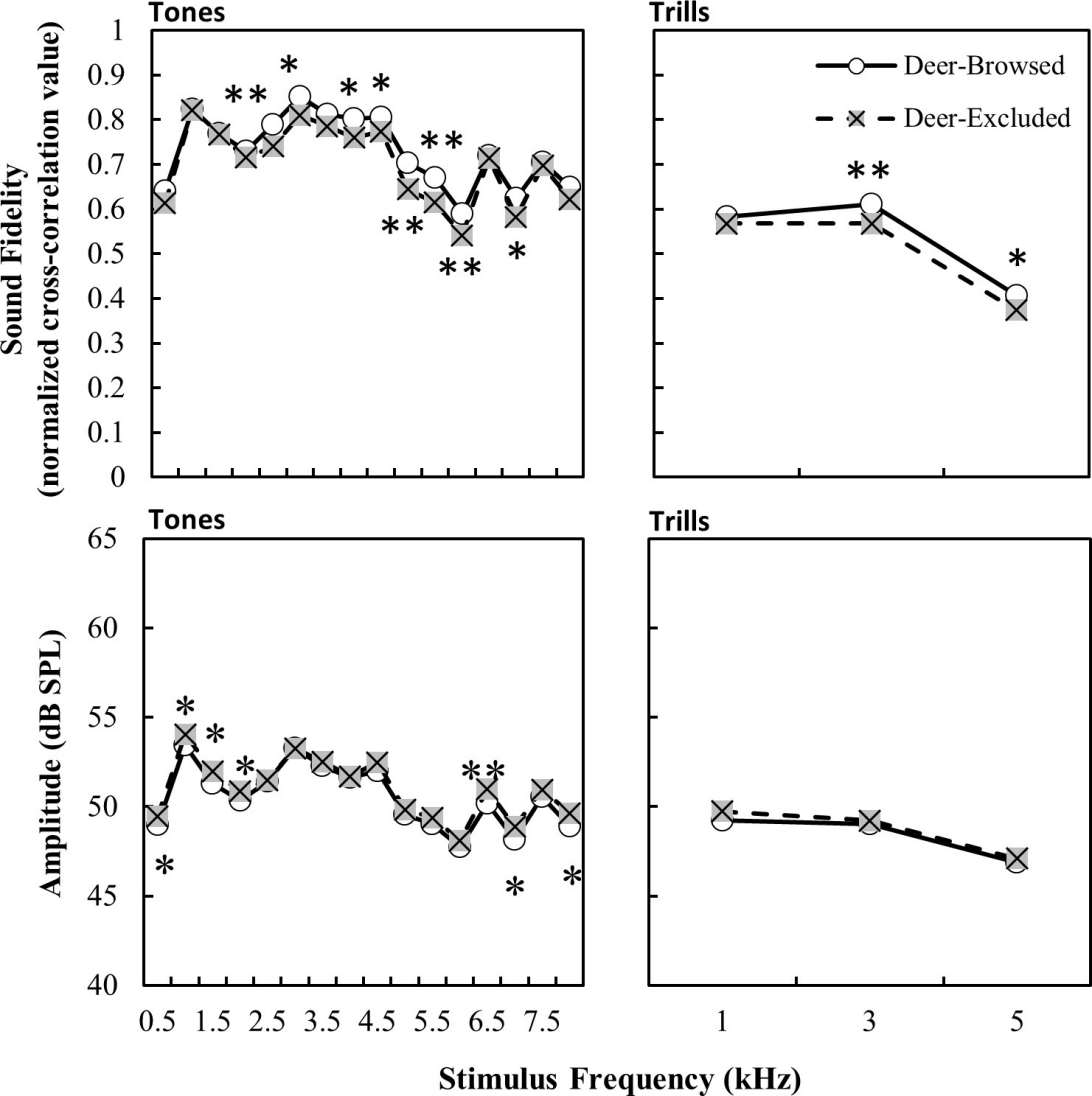
Propagation fidelity and amplitude of stimuli (lsmeans ± S.E.) as a function of stimulus frequency and browsing treatment. Significant post-hoc differences in least-square means between treatments at each frequency are denoted by * p < 0.05, ** p < 0.001.

**Fig 5:**
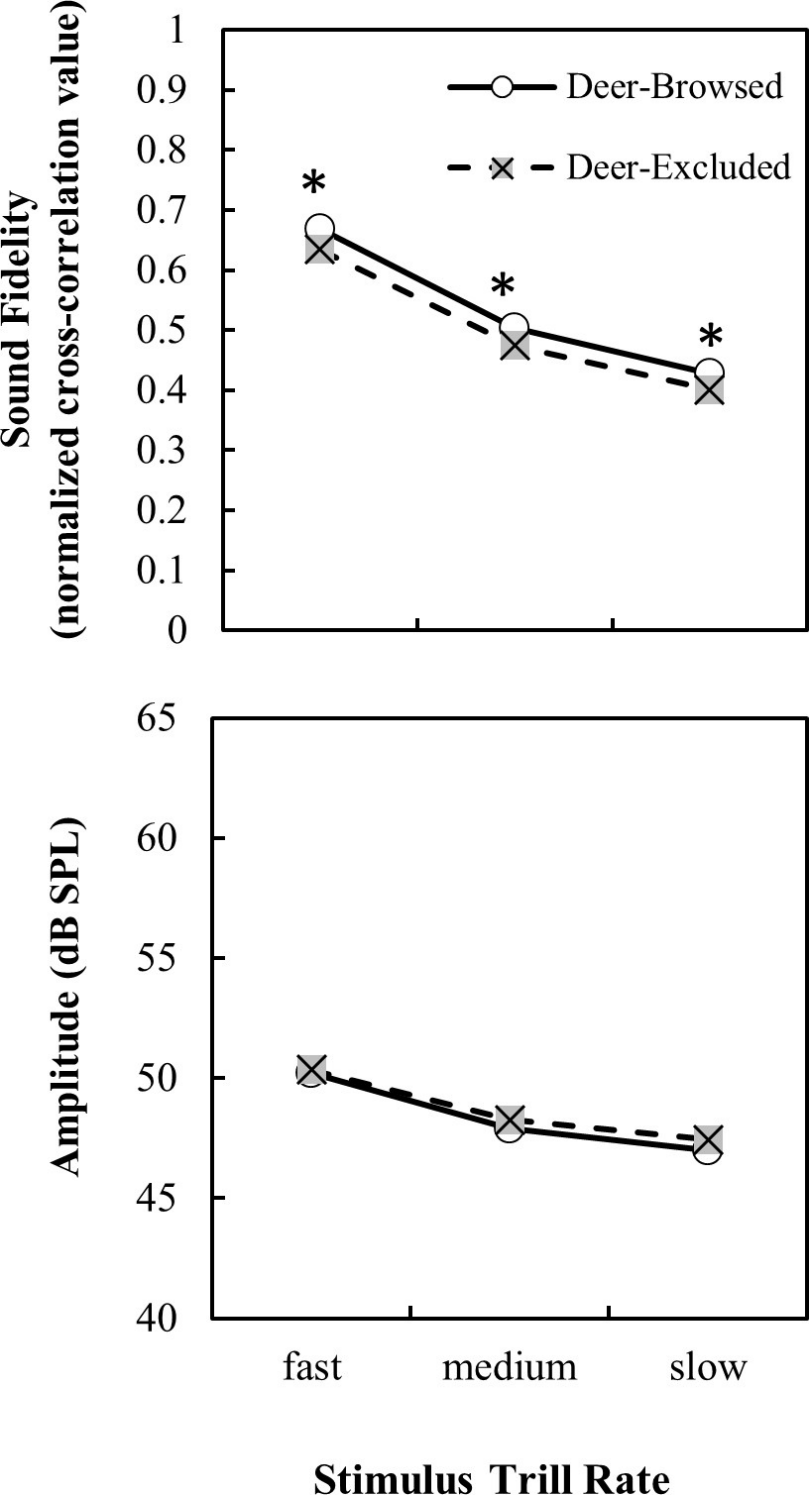
Propagation fidelity and amplitude of stimuli (lsmeans ± S.E.) as a function of trill rate and browsing treatment. Significant post-hoc differences in least-square means between treatments at each frequency are denoted by * p < 0.05, ** p < 0.001.

Generally, higher frequencies had lower fidelity while lower frequencies had higher fidelity (Table 1 and Fig 4), as is expected in a forest environment. Typically, the recordings of stimuli that overlapped with the frequency range of avian vocalizations (i.e. 2–6 kHz) differed most between browsed and unbrowsed areas (Fig 4). For trill stimuli, deer-browsed treatments had higher fidelity than deer-excluded treatments at 3 and 5 kHz (Fig 4). Trills generally had lower fidelity than other stimuli and fidelity decreased with increasing trill rate (Table 1 and Fig 5).

### Sound amplitude

Humidity and average wind speed had a significant effect on the amplitude of tones and trills, but not white noise. Additionally, temperature significantly influenced tone amplitude, but not the amplitude of trills or white noise (Table 2). The main effect of treatment did not significantly affect amplitude for any of the stimuli. As with sound fidelity, the amplitude of white noise, tones and trills decreased with increasing distance from the speaker (Table 2 and Fig 1). There was a significant treatment by distance interaction for tones, but not white noise or trills; although post-hoc analysis did not reveal significant difference in tone amplitude between treatments at any of the distances (Table 2 and Fig 1).

**Table 2:** Sound amplitude of white noise, tone, and trill stimuli.

Effect:	White Noise			Tones			Trills		
	DF	F-value	p-value	DF	F-value	p-value	DF	F-value	p-value
Temperature	1, 51.7	5.27	0.0258	1, 637	42.11	< .0001	1, 174	9.81	0.002
Humidity	1, 42.3	0.33	0.5698	1, 633	11.16	0.0009	1, 156	0.83	0.3627
Average Wind Speed	1, 102	0.22	0.6415	1, 656	4.72	0.0302	1, 251	4.56	0.0337
Treatment	1, 75.5	0.07	0.7897	1, 654	12.67	0.0004	1, 223	1.38	0.2405
Time of day	1, 64.7	14.31	0.0003	1, 645	144.13	< .0001	1, 199	27.9	< .0001
Distance	5, 285	1245.36	< .0001	5, 764	1135.88	< .0001	5, 710	384.64	< .0001
Height	2, 119	13.11	< .0001	2, 642	158.26	< .0001	2, 771	63.79	< .0001
Frequency	-	-	-	15, 4847	291.63	< .0001	2, 3105	606.54	< .0001
Trill rate	-	-	-	-	-	-	2, 2734	3970.86	< .0001
Treatment * Time of day	1, 103	0	0.9966	1, 645	2.56	0.1099	1, 247	0.33	0.5658
Treatment * Distance	5, 293	1.53	0.1812	5, 1424	3.63	0.0029	5, 1921	3.62	0.0029
Treatment * Height	2, 98.9	2.21	0.1151	2, 693	7.78	0.0005	2, 264	5.86	0.0032
Treatment * Frequency	-	-	-	15, 4852	1.28	0.2045	2, 3084	4.27	0.0141
Time of day * Frequency	-	-	-	15, 4848	17.94	< .0001	2, 2927	2.85	0.058
Distance * Frequency	-	-	-	75, 5138	9.18	< .0001	10, 2250	25.53	< .0001
Time of day * Height	+	+	+	2, 715	3.29	0.038	+	+	+
Distance * Height	10, 305	21.74	< .0001	10, 758	69.51	< .0001	+	+	+
Height * Frequency	-	-	-	30, 5027	14.23	< .0001	+	+	+
Treatment * Trill rate	-	-	-	-	-	-	2, 2855	11.57	< .0001
Distance * Trill rate	-	-	-	-	-	-	10, 2988	96.32	< .0001
Height * Trill rate	-	-	-	-	-	-	4, 2745	18.26	< .0001
Frequency * Trill rate	-	-	-	-	-	-	4, 2892	54.34	< .0001
Treatment * Time of day * Frequency	-	-	-	15, 4849	2.68	0.0005	2, 2923	0.47	0.6241
Treatment * Time of day * Height	+	+	+	2, 707	0.02	0.9785	+	+	+
Treatment * Height * Frequency	-	-	-	30, 4972	1.14	0.2683	+	+	+
Time of day * Height * Frequency	-	-	-	30, 4968	1.39	0.0751	+	+	+
Distance * Height * Frequency	-	-	-	150, 5141	3.66	< .0001	+	+	+
Treatment * Height * Trill rate	-	-	-	-	-	-	4, 2745	6.15	< .0001
Treatment * Time of day * Height * Frequency	-	-	-	30, 4969	0.65	0.9314	+	+	+

Weather variable effects, main effects, two-way interactions, three-way interactions and four-way interactions are separated by lines. Variables that are not applicable to a model (e.g. those with frequency for white noise) are indicated by (-). Non-significant higher order interactions removed prior to the final model are indicated by (+).

There was a significant effect of the treatment by height interaction for white noise and trills, but not tones (Table 2 and Fig 2). For white noise stimuli, understory recordings in the deer-excluded treatments had higher amplitude compared to deer-browsed treatments, but there were no differences at the other microphone-speaker combinations. Conversely, the trill stimuli recorded in the understory had higher amplitude in deer-browsed treatments compared to the deer-excluded treatments, although in both cases the absolute difference in amplitude was small.

The time of day at which the recordings were made also affected their amplitude. Interestingly, all stimuli types had higher amplitude earlier in the day than later in the day (Table 2 and Fig 3). There were, however, no significant interactions between treatment and time of day for any of the stimuli types (Table 2 and Fig 3). For tone and trill stimuli, there was a significant effect of frequency on amplitude, with lower frequency ranges generally having higher amplitude than higher frequency ranges (Table 2 and Fig 4). As trill-rate decreased so did the amplitude, though there were no significant interactions of trill rate with any other model variables (Table 2 and Fig 5). Lastly, in general, the amplitude for trill stimuli were lower than those of white noise and tones, likely a result of the longer inter-element periods of silence in the trilled stimuli.

## Discussion

### Vegetation structure and sound propagation

We predicted a decrease in fidelity and amplitude in deer-excluded plots, with the greatest effects on stimuli with high frequencies and greater temporal modulations (i.e. trills). Our data largely supported our predictions for stimulus fidelity, but results were mixed for stimulus amplitude. We found that playbacks had higher fidelity in deer-browsed areas when stimuli were propagated in the understory, although stimulus amplitude was largely not affected by deer-browsing treatment. Differences in vegetation structure are likely the primary driver in the differences in propagation in the two habitats, as has been found in other comparisons of sound propagation in habitats with different vegetation densities [2,5,10,47]. Given that playbacks were conducted over short distances (11 meters), the effect of atmospheric absorption on degradation is likely minimal [14,15]. However, it is possible that height-specific atmospheric conditions could have differentially affected fidelity and amplitude in the understory [9,14,15].

Deer browsing has been shown to significantly alter leaf litter composition and soil characteristics, even over very short periods of time [26,28,29]. Intense browsing regimes often result in less leaf litter and harder, more compact soils [28]. More compact soils tend to have greater reflection of sound from the surface which can lead to either signal degradation or signal enhancement; the extent to which either occurs is influenced by the angle of incidence of the sound source and atmospheric conditions near the ground [5,9,15]. Leaf litter and softer, less compact soils have higher porosity and thus greater sound deadening, particularly at higher frequencies [5,9,15]. Our results generally suggest that the density of understory vegetation is likely driving the browsing effect, with higher degrees of degradation and attenuation at lower heights and within our deer-excluded habitats, although softer soil or greater leaf litter levels could also contribute to sound attenuation. Additionally, more densely vegetated deer-excluded areas may provide better microhabitats for forest invertebrates [48,49], leading to changes in the structure of biotic background noise, which could influence fidelity and amplitude of stimuli. Furthermore, differences in vegetation structure could also interact with abiotic factors, such as weather variables, to influence sound propagation. For example, we included as random effects the weather variables of temperature, humidity and average wind speed, which did appear to affect sound propagation. Higher wind speeds could have influenced the fidelity and attenuation of signals through the introduction of temporal modulations or by masking from wind noise, or by interacting with vegetation to create higher levels of background noise [8,50]. Background noise levels may be influenced by deer browsing when we consider large areas of forest and these differences in background noise levels could be important for communication. However, the differences in sound propagation which we found are unlikely to be confounded by background noise levels due to the proximity of our paired plots. This was corroborated by our analysis of background noise, which suggested no differences between treatments. We found that higher frequencies suffered more degradation and attenuation than lower frequencies across both habitat types. Furthermore, the difference in fidelity between browsed and unbrowsed areas was most pronounced at high frequencies. This was expected as, generally, high frequencies are subject to greater absorption by the atmosphere [14,15,51] and scattering by physical objects [2,12]. Stimuli with more temporal variation (trills) were also more degraded. Within trill stimuli, faster trills suffered less degradation than slower trills. This was surprising, as most studies have shown the opposite, with faster trills suffering most degradation, due to higher degrees of reverberation [10,44,52,53]. However, we measured fidelity and amplitude of the entire trill, including the silent intervals between elements, rather than individual elements. Slower trills have longer periods of “silence” between each individual tone element, and the overall sound structure is thus more likely to be subject to interference from background noise, although our background noise levels were relatively constant.

Finally, we found that the time of day also influenced sound propagation. Sound recordings made earlier in the day had lower sound fidelity than those made later in the day, and the effect of deer browsing treatment was more pronounced earlier in the day. Amplitude, however, had a reverse trend, being lower later in the day compared to earlier in the day. This was surprising, as pre-dawn hours typically see cooler temperatures, drier conditions, and less air turbulence, and these atmospheric conditions allow for efficient transmission of sound, and less degradation to sound signals [54]. We first thought that changes across the day could be due to dawn chorus-related changes in background noise levels. However, two lines of evidence suggest that this is not the primary driver of these differences in propagation. First, we found (anecdotally) that during the course of our first playback the chorus notably subsided from our pre-dawn arrival at the study site. Second, we found no difference in background noise levels nor the spectral composition of the background noise across the two time periods, suggesting background noise differences are not the primary drivers of time-related propagation differences. However, our background noise levels were limited in scope and a greater diversity of recordings may provide a more detailed look at the spectral composition of the noise across the day. A larger sample size of background noise may reveal time of day related differences across the two treatments. There are a number of factors that could also be contributing to the time of day effects, including weather variables or other atmospheric conditions [2,8]. However, we did not conduct fine-scale analyses of how these variables behaved within our treatment plots, but future studies could tease apart how various biotic and abiotic aspects of a habitat are related to sound propagation in the context of deer browsing-induced habitat changes.

### Signal propagation, habitat choice and signal evolution

Our results show that signal propagation is affected by deer browsing, likely through changes in vegetation structure. We have a clear understanding of the relationship between the physical environment and sound propagation [2,4] and that browsing-mediated changes in forest vegetation structure have been linked with declines in various species of birds [36–38]. However, the connection between deer browsing and vocal communication is less clear. Although we used relatively simple stimuli, rather than vocalizations, we believe that the changes we observed could have implications for avian communication, community structure and signal evolution. In particular, our results are best suited to understand how deer browsing could affect behaviors that involve short-distance communication. For example, during the breeding season a variety of behaviors involve communication over short distances, including courtship behaviors, nest guarding and other aggressive encounters (e.g. soft songs in song sparrows or gargle calls in black-capped chickadees), and parent-offspring interactions [40–42,55–57]. Furthermore, there are additional short-distance communication signals, such as those involved in foraging or predator defense, which may also be impacted by changes in signal propagation characteristics [39,58,59]. Changes that enhance the propagation of communication signals could be beneficial in some circumstances (enhanced flock cohesion or anti-predator warnings). However, in other cases increased signal propagation could be detrimental, as it may enhance the ability of eavesdroppers (e.g. predators or conspecific competitors) to gather information about nest locations, mating opportunities, or fighting ability [60,61]. Additionally, some species are known to select breeding sites based on the acoustic characteristics of that habitat [62–64]; thus deer-induced changes in sound propagation may alter the avian community structure through changes in habitat acoustic characteristics. Although the behavioral implications of deer browsing on signal propagation are not yet clear, there is ample opportunity for investigation.

Over the course of decades, sustained levels of deer browsing pressure and the resulting understory vegetation loss and declines in tree recruitment could lead to increasingly open habitats [19,21,27]. Concomitantly, changes in acoustic signal structure are likely to occur across or within species, and could result from plasticity, cultural evolution, or local adaptation [65–67]. In many cases, a combination of factors may influence differences in vocal production across populations. Frogs and birds have been shown to plastically alter vocalizations in response to changes in the signal propagation properties of their habitats. For example, in urban areas, animals can shift the timing, increase the duration, or modify the amplitude of their vocalizations to avoid competing with anthropogenic noise [68–71]. Individuals of some species may also plastically adjust their vocalizations in response to the vocalizations of other species, resulting in acoustic niche partitioning differing across habitats based on community structure [59,72]. Plastic changes in vocal behavior in response to anthropogenic noise have largely been investigated over minutes or hours [68,73]; while habitat changes associated with increased deer browsing or implementation of a deer management program are likely to change the habitat over the course of months or years. Animals living in these habitats may plastically change their vocalizations over the course of their lifetime to best take advantage of the propagation properties of their habitat.

Animal vocalizations may also change as a function of habitat characteristics through cultural evolution. There is some evidence to suggest that preferential learning of efficiently propagated vocalizations may drive cultural evolution of these signals. Young birds tend to learn vocalizations from the least degraded model signals [74–77]. The least degraded model may differ across habitats if the propagation characteristics of the environment differs. For example, white-crowned sparrows (*Zonotrichia leucophrys*) in urban habitats, preferentially learn songs that minimize masking from background noise, leading to the cultural evolution of higher frequency songs in the populations of these habitats. Meanwhile, for white-crowned sparrow populations in more rural habitats, the same trend is not observed [56–58]. Less degraded signals presumably improve the transfer of information between a sender and receiver [78–80]. Preferential learning of optimal signals could lead to these versions of signals becoming dominant in a given acoustic environment [3,74,65]. If signals propagate (and thus are degraded) differently in deer-browsed and unbrowsed habitats, this could lead to instances of preferential learning and thus cultural evolution. There is considerably less work on the cultural evolution of calls, but some short distance calls are known to be shaped by cultural evolution, such as the gargle calls of black-capped chickadees [81,82], and could thus potentially be influenced by the propagation characteristics of the environment.

Finally, vocalizations may change as a function of habitat characteristics due to local adaptation that favors signal efficacy (i.e. Acoustic Adaptation Hypothesis; 3,4). The acoustic adaptation hypothesis has received mixed support but is widely accepted and appears to hold across a variety of habitats [53,65,75]. Selection pressures often favor acoustic signals that minimize degradation and attenuation, thus enhancing the propagation of signals and optimizing communication in a given environment [3,4,74]). Changes to the sound propagation characteristics of an environment could, therefore, contribute to the local adaptation of vocalizations in resident populations over time [3,5,6,83]. Alternatively, acoustic signals that are rendered maladaptive by changes in sound propagation characteristics of an environment could contribute to changes in species composition or abundance as individuals move to habitats in which their vocalizations are better suited [62–64]. Although the relationship between animal communication and deer-induced changes in sound propagation is speculative at this time, our results do suggest interesting avenues for future work on signal evolution in populations of songbirds and other taxa that acoustically communicate in forests that are subject to different levels of deer browsing.

## Conclusions

Overall, we found that sound propagation is altered in deer-browsed habitats. Our study only looked at sound propagation over short distances and thus our results are most applicable to short distance communication signals, such as those used in courtship, agonistic encounters, or parent-offspring communication. We used artificial sound stimuli to assess how basic sound propagation characteristics were influenced by deer browsing. However, bird songs and calls vary greatly in their structure and different species may be affected differently by deer browsing. Thus, future studies investigating species-specific propagation patterns would be very interesting. Altogether, our results suggest that there may be a surprising connection between deer-induced habitat alteration and animal communication. Future work should investigate the differences in soundscapes among habitats with different levels of deer browsing, as well as investigate the production and reception of acoustic communication signals in avian populations that are found in habitats with different levels of deer browsing. Furthermore, it may be informative to investigate sound propagation over greater distances that would be representative of long distance communication.

## Supporting information

S1 Table Relative sound amplitude of white noise, tone, and trill stimuli. Effects and interactions reported include those of significance as well as those which are not significant but are of value for comparisons across model types. The "-" symbol denotes that the effect is not applicable to the model. The "+" symbol denotes that effect is not significant in the current model. All effects were initially included in full models. Non-significant higher order interactions were subsequently removed from models according to p-values and resulting AIC values.
(DOCX)

S2 Table Background noise level by time of day and browsing treatment. Background noise level was determined for the entire frequency range of the stimuli (all: 400–8100 Hz) and in 200 Hz bands every 500 Hz from 500 to 8000 Hz (in other words each center frequency of the tones ± 100 Hz). Noise levels were determined from 6 minutes of background noise recordings during the playback trials. The noise was then filtered using a Hahn passband filter in PRAAT with a smoothing of 50 Hz. Sound intensity was then found for each filtered stimulus with the Get Intensity function in PRAAT.
(DOCX)

S1 Fig Photo examples of microphone and speaker arrangement, as well as a comparison of deer-browsed and deer-excluded plots. (DOCX)

S2 Fig Sound attenuation (lsmeans ± S.E.) of acoustic stimuli in deer-browsed and deer-excluded plots as a function of distance. Here attenuation was measured as the difference between the amplitude of the propagated stimulus at 1m and each subsequent distance (3-11m).
(DOCX)

S3 Fig Sound attenuation (lsmeans ± S.E.) of acoustic stimuli in deer-browsed and deer-excluded plots as a function of stimulus frequency. Here attenuation was measured as the difference between the amplitude of the propagated stimulus at 1m and each subsequent distance (3-11m).
(DOCX)

S1 Appendix Raw data. Cross-correlation (CC) and attenuation (dB) data for tones, trills and white noise stimuli, and amplitude (dB) data for background noise.
(XLS)
