# Supplementary material for: Deer browsing alters sound propagation in temperate deciduous forests
Source: PLoS One. 2019 Feb 13;14(2):e0211569. doi: 10.1371/journal.pone.0211569 (PMC6373924; doi:10.1371/journal.pone.0211569)
Supplement: S1 Table — Effects and interactions reported include those of significance as well as those which are not significant but are of value for comparisons across model types. The "-" symbol denotes that the effect is not applicable to the model. The "+" symbol denotes that effect is not significant in the current model. All effects were initially included in full models. Non-significant higher order interactions were subsequently removed from models according to p-values and resulting AIC values. (DOCX) [file pone.0211569.s002.docx]

**S1 Table. Relative Sound Amplitude of white noise, tone, and trill stimuli.**

| Effect: | White Noise | | | Tones | | | Trills | | |
| --- | --- | --- | --- | --- | --- | --- | --- | --- | --- |
|  | DF | F-value | p-value | DF | F-value | p-value | DF | F-value | p-value |
| Temperature | 1, 46.1 | 0.15 | 0.7042 | 1, 659 | 0.24 | 0.6242 | 1, 182 | 21.26 | <.0001 |
| Humidity | 1, 40.5 | 0.09 | 0.7637 | 1, 655 | 0.2 | 0.6555 | 1, 169 | 1.8 | 0.1817 |
| Average Wind Speed | 1, 74.5 | 2.94 | 0.0908 | 1, 673 | 14.03 | 0.0002 | 1, 233 | 3.87 | 0.0503 |
| Treatment | 1, 67.5 | 1.61 | 0.2084 | 1, 672 | 10.19 | 0.0015 | 1, 224 | 12.01 | 0.0006 |
| Time of day | 1, 55.7 | 0.19 | 0.6633 | 1, 665 | 0.21 | 0.6442 | 1, 200 | 26.31 | <.0001 |
| Distance | 4, 227 | 378.73 | <.0001 | 4, 764 | 400.74 | <.0001 | 4, 559 | 73.58 | <.0001 |
| Height | 2, 232 | 340.83 | <.0001 | 2, 667 | 2074.99 | <.0001 | 2, 587 | 6.41 | 0.0018 |
| Frequency | - | - | - | 15, 3993 | 105.67 | <.0001 | 2, 2629 | 2760.46 | <.0001 |
| Trill rate | - | - | - | - | - | - | 2, 2172 | 512.58 | <.0001 |
| Treatment * Time of day | 1, 73.1 | 0.02 | 0.9012 | 1, 665 | 1.4 | 0.2373 | 1, 228 | 0.64 | 0.4256 |
| Treatment * Distance | 4, 248 | 2.04 | 0.0895 | 4, 1162 | 4.23 | 0.0021 | 4, 1609 | 3.24 | 0.0117 |
| Treatment * Height | 2, 92.8 | 2.33 | 0.1032 | 2, 704 | 14.43 | <.0001 | 2, 278 | 6.77 | 0.0013 |
| Treatment * Frequency | - | - | - | 15, 3997 | 2.66 | 0.0005 | 2, 2555 | 25.39 | <.0001 |
| Time of day * Frequency | - | - | - | 15, 3994 | 8.75 | <.0001 | 2, 2440 | 33.59 | <.0001 |
| Distance * Frequency | - | - | - | 60, 4232 | 3.76 | <.0001 | 8, 1806 | 7.63 | <.0001 |
| Time of day * Height | + | + | + | 2, 718 | 7.61 | 0.0005 | + | + | + |
| Distance * Height | + | + | + | 8, 756 | 6.85 | <.0001 | + | + | + |
| Height * Frequency | - | - | - | 30, 4155 | 44.61 | <.0001 | + | + | + |
| Treatment * Trill rate | - | - | - | - | - | - | 2, 2246 | 0.09 | 0.9102 |
| Distance * Trill rate | - | - | - | - | - | - | 8, 2513 | 43.01 | <.0001 |
| Height * Trill rate | - | - | - | - | - | - | 4, 2188 | 5.67 | 0.0002 |
| Frequency * Trill rate | - | - | - | - | - | - | 4, 2393 | 93.74 | <.0001 |
| Treatment * Time of day * Frequency | - | - | - | 15, 3995 | 2.41 | 0.0017 | 2, 2439 | 6.65 | 0.0013 |
| Treatment * Time of day * Height | + | + | + | 2, 713 | 4.42 | 0.0124 | + | + | + |
| Treatment * Height * Frequency | - | - | - | 30, 4119 | 1.4 | 0.0737 | + | + | + |
| Time of day * Height * Frequency | - | - | - | 30, 4117 | 4.56 | <.0001 | + | + | + |
| Distance * Height * Frequency | - | - | - | 120, 4249 | 1.64 | <.0001 | + | + | + |
| Treatment * Height * Trill rate | - | - | - | - | - | - | 4, 2187 | 5.3 | 0.0003 |
| Treatment * Time of day * Height * Frequency | - | - | - | 30, 4118 | 2.28 | <.0001 | + | + | + |

Effects and interactions reported include those of significance as well as those which are not significant but are of value for comparisons across model types. The "-" symbol denotes that the effect is not applicable to the model. The "+" symbol denotes that effect is not significant in the current model. All effects were initially included in full models. Non-significant higher order interactions were subsequently removed from models according to p-values and resulting AIC values.
