# Supplementary material for: Deer browsing alters sound propagation in temperate deciduous forests
Source: PLoS One. 2019 Feb 13;14(2):e0211569. doi: 10.1371/journal.pone.0211569 (PMC6373924; doi:10.1371/journal.pone.0211569)
Supplement: S2 Table — Background noise level was determined for the entire frequency range of the stimuli (all: 400–8100 Hz) and in 200 Hz bands every 500 Hz from 500 to 8000 Hz (in other words each center frequency of the tones ± 100 Hz). Noise levels were determined from 6 minutes of background noise recordings during the playback trials. The noise was then filtered using a Hahn passband filter in PRAAT with a smoothing of 50 Hz. Sound intensity was then found for each filtered stimulus with the Get Intensity function in PRAAT. (DOCX) [file pone.0211569.s003.docx]

**S2 Table. Background noise level by time of day and browsing treatment.**

|  | Deer Excluded | | | | Deer Browsed | | | |
| --- | --- | --- | --- | --- | --- | --- | --- | --- |
|  | Early | | Late | | Early | | Late | |
| Frequency (Hz) | Mean | ± S.D. | Mean | ± S.D. | Mean | ± S.D. | Mean | ± S.D. |
| all | 36.05 | ± 2.85 | 39.1 | ± 4.08 | 37.51 | ± 3.38 | 37.05 | ± 4.56 |
| 500 ± 100 | 32.63 | ± 3.54 | 32.16 | ± 5.38 | 27.58 | ± 3.67 | 28.95 | ± 1.51 |
| 1000 ± 100 | 25.23 | ± 2.98 | 26.00 | ± 4.21 | 20.93 | ± 3.21 | 25.32 | ± 3.47 |
| 1500 ± 100 | 18.49 | ± 3.13 | 21.33 | ± 3.87 | 16.95 | ± 3.04 | 21.21 | ± 3.46 |
| 2000 ± 100 | 17.73 | ± 5.50 | 17.19 | ± 4.41 | 16.61 | ± 5.09 | 21.22 | ± 4.09 |
| 2500 ± 100 | 17.77 | ± 6.54 | 16.70 | ± 5.35 | 20.23 | ± 5.01 | 24.82 | ± 4.95 |
| 3000 ± 100 | 18.91 | ± 7.45 | 16.59 | ± 6.47 | 21.01 | ± 5.49 | 24.33 | ± 6.43 |
| 3500 ± 100 | 18.00 | ± 4.40 | 15.19 | ± 6.22 | 20.54 | ± 5.06 | 23.12 | ± 6.8 |
| 4000 ± 100 | 15.43 | ± 7.20 | 13.49 | ± 6.36 | 17.74 | ± 6.44 | 21.24 | ± 6.95 |
| 4500 ± 100 | 13.55 | ± 6.92 | 11.66 | ± 5.58 | 13.26 | ± 8.09 | 17.78 | ± 7.27 |
| 5000 ± 100 | 12.27 | ± 7.58 | 9.84 | ± 3.35 | 11.02 | ± 9.75 | 17.36 | ± 8.08 |
| 5500 ± 100 | 10.27 | ± 8.27 | 7.60 | ± 2.31 | 7.98 | ± 9.2 | 15.30 | ± 7.81 |
| 6000 ± 100 | 9.50 | ± 6.63 | 6.73 | ± 1.65 | 6.63 | ± 7.95 | 12.90 | ± 6.38 |
| 6500 ± 100 | 8.51 | ± 5.99 | 4.88 | ± 1.76 | 5.70 | ± 7.08 | 13.12 | ± 4.7 |
| 7000 ± 100 | 8.54 | ± 5.06 | 4.87 | ± 4.32 | 4.93 | ± 6.98 | 11.93 | ± 4.04 |
| 7500 ± 100 | 8.10 | ± 4.98 | 3.84 | ± 4.88 | 3.65 | ± 7.16 | 10.76 | ± 5.75 |
| 8000 ± 100 | 4.45 | ± 5.53 | 0.43 | ± 3.29 | 0.93 | ± 6.77 | 7.28 | ± 4.33 |

Background noise level was determined for the entire frequency range of the stimuli (all: 400-8100 Hz) and in 200 Hz bands every 500 Hz from 500 to 8000 Hz (in other words each center frequency of the tones ± 100 Hz). Noise levels were determined from 6 minutes of background noise recordings during the playback trials. The noise was then filtered using a Hahn passband filter in PRAAT with a smoothing of 50 Hz. Sound intensity was then found for each filtered stimulus with the Get Intensity function in PRAAT.
