# Supplementary material for: Deer browsing alters sound propagation in temperate deciduous forests
Source: PLoS One. 2019 Feb 13;14(2):e0211569. doi: 10.1371/journal.pone.0211569 (PMC6373924; doi:10.1371/journal.pone.0211569)
Supplement: S1 Fig — (DOCX) [file pone.0211569.s001.docx]

**
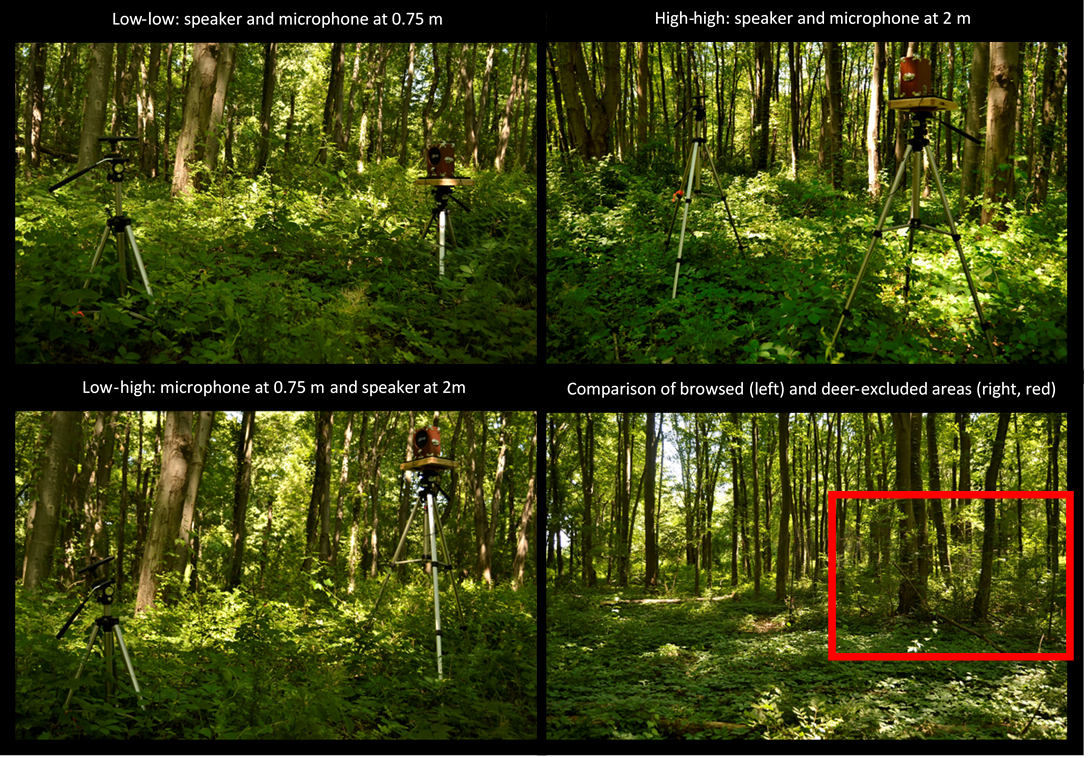
****S1 Fig.** Examples of microphone and speaker arrangement, as well as a comparison of deer-browsed and deer-excluded plots.
