# Supplementary material for: Deer browsing alters sound propagation in temperate deciduous forests
Source: PLoS One. 2019 Feb 13;14(2):e0211569. doi: 10.1371/journal.pone.0211569 (PMC6373924; doi:10.1371/journal.pone.0211569)
Supplement: S3 Fig — Here attenuation was measured as the difference between the amplitude of the propagated stimulus at 1m and each subsequent distance (3-11m). (DOCX) [file pone.0211569.s005.docx]

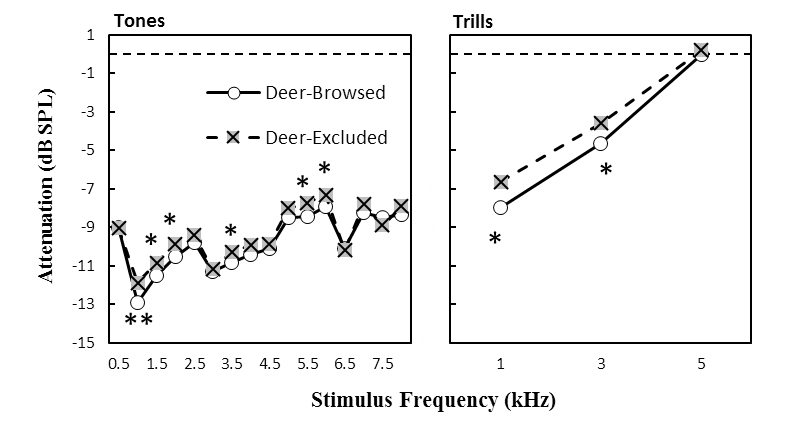


**S3 Fig.** Sound attenuation (lsmeans ± S.E.) of acoustic stimuli in deer-browsed and deer-excluded plots as a function of stimulus frequency. Here attenuation was measured as the difference between the amplitude of the propagated stimulus at 1m and each subsequent distance (3-11m).
